# NEuronMOrphological analysis tool: open-source software for quantitative morphometrics

**DOI:** 10.3389/fninf.2013.00002

**Published:** 2013-02-14

**Authors:** Lucia Billeci, Chiara Magliaro, Giovanni Pioggia, Arti Ahluwalia

**Affiliations:** ^1^Institute of Clinical Physiology (IFC), National Research Council of Italy (CNR)Pisa, Italy; ^2^Interdepartmental Research Center “E. Piaggio,” Faculty of Engineering, University of PisaPisa, Italy

**Keywords:** image processing, software, morphometrics, 3-way PCA, neurons

## Abstract

Morphometric analysis of neurons and brain tissue is relevant to the study of neuron circuitry development during the first phases of brain growth or for probing the link between microstructural morphology and degenerative diseases. As neural imaging techniques become ever more sophisticated, so does the amount and complexity of data generated. The NEuronMOrphological analysis tool NEMO was purposely developed to handle and process large numbers of optical microscopy image files of neurons in culture or slices in order to automatically run batch routines, store data and apply multivariate classification and feature extraction using 3-way principal component analysis (PCA). Here we describe the software's main features, underlining the differences between NEMO and other commercial and non-commercial image processing tools, and show an example of how NEMO can be used to classify neurons from wild-type mice and from animal models of autism.

## Introduction

It is well-known that even at the microstructural level, neuronal morphology is important for higher level brain function (White, 2007; Brown et al., 2008). For example, the analyses of neural structure and neural organization over time is crucial for the study of neuron circuitry development during the first phases of brain growth or for probing the link between cell morphology and degenerative diseases (Zhao et al., 2007). Classically neuron morphology is investigated using stained and fixed dissociated cultures or slices (Cajal, 1952; Golgi, 1989; Kapfhammer, 2004). Most investigators now use imaging techniques such as confocal, 2-photon and super-resolution microscopy, and several reports describe neural growth dynamics using fluorophores such as tracker dyes or calcium sensitive dyes (Arai et al., 1999; Blinder et al., 2005; Volman et al., 2005). Genetically encoded probes are also becoming commonplace, and are an extremely useful tool for analyzing neural cell morphology, although efforts are also dedicated toward cell signaling particularly at the synaptic level (Knopfel et al., 2006). Several reports use techniques such as 2-photon or confocal microscopy on organotypic brain slices, which enable short term (10 min) recordings of calcium dynamics through the use of Ca^2+^ specific dyes or very high magnification static analysis of dendritic spine distribution (O'Brien and Unwin, 2006; Lillis et al., 2008).

As the spatial resolution and acquisition frequency of imaging techniques increases, so has our ability to generate huge quantities of data on neuronal morphometry and dynamics. However, it is quite often time consuming and difficult to batch process image files and most of the digital image processing techniques such as segmentation and feature extraction, which have changed little over the past few decades, still require a large degree of pre-processing and image manipulation. Furthermore, the manual quantification of neuronal morphology is very labor-intensive and is prone to observer bias. Not only lack of consistency within an individual observer, but also variance between different observers can reduce the level of reproducibility (Schmitz et al., 2011).

In the last 20 years many open-source or commercial tools have been implemented for automatically and consistently quantifying neuronal morphology through image processing (see section “Discussion”). However, it is still not possible to perform complete morphological analyses of a collection of images using a unique tool. More importantly, no software is designed to conduct time-dependent quantification of neuron morphometry during growth or differentiation, nor is it possible to perform in-depth statistical analyses on a collection of related images. Finally, as far as data analysis is concerned, most studies and software based methods which describe neuron morphometrics use very simple statistical tests such as the *t*- or *f*-tests, which are often unsuitable for the study and classification of complex multivariate data.

To overcome these limitations, we have designed a novel user-friendly software, NEMO (acronym of “NEuron MOrphological tool”) for batch processing of neuron images for dynamic morphometric analysis. Time lapse sequences of neurons or brain slices can be processed and analyzed sequentially with automatic data storage in the form of plots and matrices containing morphometric data. Subsequently the matrices can be analyzed using 3-way principal component analysis (PCA), which enables the organization of multivariate datasets into groups, thus facilitating the interpretation of complex and large groups of data.

In this paper, we describe the unique features of NEMO and illustrate an example of how the software can be used to distinguish and classify neurons using multivariate analysis. In particular, the software was used for morphological studies of Purkinje neurons in culture and for comparison of two different genotypes, L7GFP WT (wild-type) mice and the L7GFP/EN2+/− knock-out. Purkinje cells from the LF7GFP strain express GFP (green fluorescent protein), while the engrailed gene EN2 is associated with autism, and is known to cause morphological alterations at the cerebellar level (Kuemerle et al., 2007).

## NEMO development and implementation

NEMO is an open-source software, which can be downloaded from http://www.centropiaggio.unipi.it/software (Billeci et al., 2011). The software was developed in Matlab® code (The MathWorks-sTM, Inc, USA) and performs micro-structural and quantitative analysis from 2D images of neurons and organotypic brain slices. It has a user friendly graphical user interface (GUI) environment with pull down menus for image pre-processing, quantitative morphological and topological analysis, automatic counting of neurons in an image, plotting of the features extracted and statistical analysis by 3-way PCA.

Prior to performing a morphometric analysis of neurons in culture, individual cells are skeletonized: the cell is reduced to a binary image with a one pixel thick line. After the skeletonization process, images of single neurons are analyzed with the morphological analysis tool. Alternatively images of organotypic brain slices can be processed through the automatic counting and a topological analysis tool for the extraction of relevant metrical variables.

All the measurements extracted can be then analyzed by the graphical and statistical tools implemented in the software. The tools and functions available in NEMO are summarized in Table 1.

**Table 1:** **Tools and functions implemented in NEMO**.

**Tools**	**Functions**
**LOADING IMAGES**	
Image processing	Gaussian filter
	Uniform background
	Grayscale conversion
	Thresholding
	Morphological operations
	Boundary detection
	Region filling
	Skeletonization
Morphological analysis	**Intersections**
	**Critical radius (✓)**
	**Maximum number of intersections (✓)**
	Schoenen ramification index
	Regression coefficient for log-log Sholl method (✓)
	Regression coefficient for semi-log Sholl method (✓)
	Correlation coefficient for log-log Sholl method
	Correlation coefficient for semi-log Sholl method
	Determination ratio
	**Minimum length vectors**
	**Angles between minimum length vectors**
	**Minimum pathway (✓)**
	**Radial extension (✓)**
	Cone angle (✓)
	**Soma area (✓)**
	Fractal dimension (✓)
**Neuron count**	Fluorescent/non-fluorescent cells
	Unique/separate plot
Topological analysis	Analysis on slice
	Plotting
**Plot morphological variables**	Datamatrix creation
	Plotting
3-way PCA	Datamatrix creation
	Multivariate analysis

In the morphological analysis section, (✓) indicates the variables chosen for 3-way PCA analysis. For the sake of comparison features not available in NeuroLucida are highlighted in bold.

## NEMO: an outline

### Loading an image in NEMO

In order to perform batch operations on images they are first renamed with appropriate labels. NEMO uses the properties indicated in the name to extract information about the neurons represented in the image. Image names have to be structured in the following way:

ImageType_CultureNumber_CellNumber_PhotographDay_CellAge

where:

- *ImageType*: “p” for “photograph,” “b” for “binary” and “s” for “skeleton.” NEMO automatically saves binary images and skeletons using these labels.
- *CultureNumber*: progressive number identifying the culture.
- *CellNumber*: within each culture, a progressive number identifying a single neuron.
- *PhotographDay*: progressive number indicating the number of days since the start of image acquisition.
- *CellAge*: progressive number indicating the age in days of the neuron pertaining to *CellNumber*.

For example, b_01_01_02_03 refers to the binary image of the first neuron belonging to the first culture, photographed since the second day of life in culture and which is at its third day of life in culture. All the images from a single experiment are stored in a folder for batch processing.

Once the image has been renamed it can be loaded into NEMO.

### Image pre-processing and neuron reconstruction

**Figure 1:**
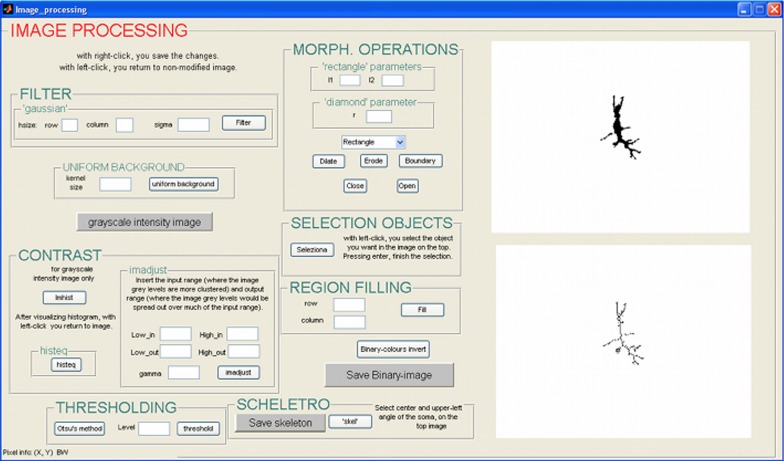
**GUI for image pre-processing**.

Images can be processed using a semi-automatic procedure, implemented in a Matlab editor, or though a GUI (Figure 1) that allows more freedom in the choice of parameters. There is no single best approach: it is necessary to find a trade-off between customization and automation of the analysis and the choice depends on the quality of the image. Alternatively images can be processed using a variety of semi-automated software tools, or by hand, and then imported into NEMO for multivariate analysis.

While in the editor there is a unique program flux, which is interrupted only when user has to insert some parameters, the GUI is organized in subroutines, so the user can run the same algorithms repeatedly, or can delete the result of the last operation. The techniques used for skeletonization are standard and therefore not described in detail. Briefly the functions implemented within this tool are:

- *Filter*: Gaussian filter, useful to minimize small variations in color and to prevent the detection of non-existent and unwanted contours;
- *Uniform background*: an algorithm that estimates the brightness of the background, and then reduces it to a single intensity value;
- *Grayscale conversion*: conversion of RGB image to grayscale intensity image;
- *Enhancing contrast*: histogram stretching or histogram equalization.
- *Thresholding*: two different thresholding algorithms have been implemented. The gray level can be chosen by the user, or the “Otsu method” scan be used to automatically find a threshold level, T.
- *Morphological operations*: dilating, eroding, opening and closing morphological operations as well as boundary detection and region filling;
- *Object selection*: the for labeling of connected objects;
- *Skeletonization*: reduces the shape of the neuron to a skeleton. A thinning algorithm has been used. Although it is accurate in dendrite reconstruction, it is not very precise in soma recognition. For this reason, the user needs to perform an additional manual operation consisting in indicating the soma center and the upper-left vertex of the soma with a mouse click.

Since neuron reconstruction is notoriously difficult, particularly with low resolution images, to compare NEMO with ImageJ, we selected representative images from different sources to reconstruct neuron skeletons.

### Neuron counting

This operation can be performed on images representing cultures of neurons or slices. When loaded in this tool, images need to be named and organized with the same criteria explained above.

The basic idea of the algorithm is to obtain a binary image, and then to count the white objects present in the image, which represent the cells. Thus it is not necessary to reconstruct the cell outline, and is better to have only somas in the image in order to avoid the counting of several segments of the same cell separated by thresholding. The algorithm works recursively: when the user selects an image in a specific folder, the algorithm automatically counts the neurons represented in all the images in that folder. So, number of cells in a slice or culture is rated for all the days in which it is photographed. Plots representing the development of cell counts over multiple images can be visualized. The user can choose between two types of plots:

- Plot separate: the user needs to choose the array to be analyzed and the algorithm automatically reports a graph with cell number as a function of days;
- Plot unique: with multi-selection, the user can choose several arrays to be analyzed. The algorithm automatically reports a unique plot with cell number as a function of days for all the selected arrays.

### Morphological analysis

**Figure 2:**
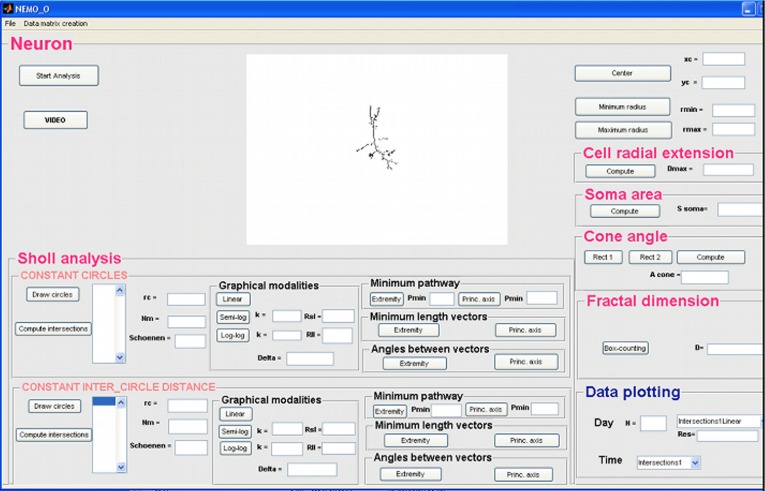
**GUI for morphological analysis of a single neuron**.

Once the neuron skeleton is obtained, the user can run the morphological analyses, which are implemented, in a specific GUI (Figure 2). The GUI calculates a vast array of morphological parameters and this feature is unique to NEMO as other software packages only output a small subset of these (see section “Discussion”).

The morphological analysis allows the contribution of metrical features and their evolution over time to be studied quantitatively. Using the GUI, the metrical features relevant to the cell's structure and morphology are extracted. The morphological variables were chosen from those generally reported in the literature (Uylings and van Pelt, 2002; Shefi et al., 2005) and divided into two groups, local and global variables. The former are referred to the dendritic tree, while the latter variables, such as radial extension, soma area, cone angle, and fractal dimension, are related to the whole cell structure. Following the Sholl method (Sholl, 1953), each cell skeleton is circumscribed a coordinate system consisting of a series of concentric circles centered on the soma. Local variables were extracted by counting the number of intersections between each circle and the cell's dendrites. A list of variables assessed with this tool is given in Table 1.

The tool works recursively: the user selects the image representing the first photograph of a neuron, and the algorithm automatically analyzes all the images in the folder, representing the same neuron photographed in successive days of culture. The variables measured with this tool are 177. Each morphological parameter is automatically saved in a specific position in a three-dimensional datamatrix, whose dimensions are 1 × 177 × No. of days of culture.

The datamatrix is automatically named with a similar criterion to that used for the images. Comparisons between NEMO and NeuronMorpho/ImageJ in analyzing common neuron morphological features and in Sholl analysis were conducted to test NEMO's performance.

### Topological analysis

This tool has been implemented for the analysis of the topological organization of organotypic brain slices and allows the contribution of metrical features and their evolution to be studied quantitatively.

**Figure 3:**
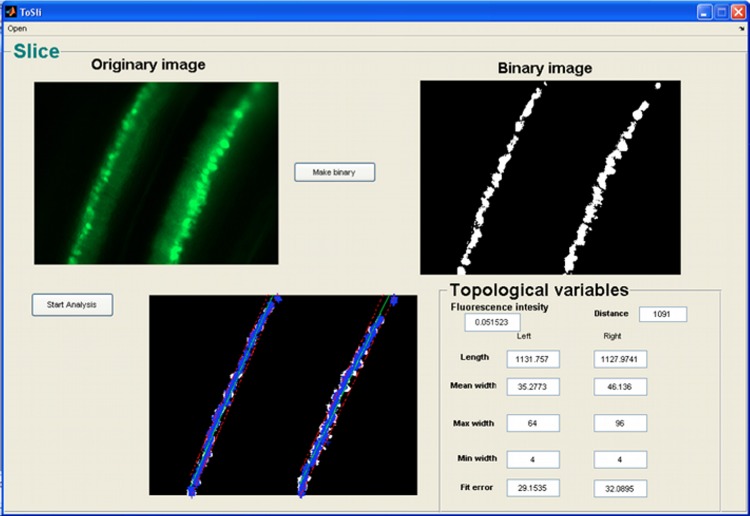
**GUI for topological analysis of organotypic brain slices**.

Segments of organotypic brain slices must be photographed at regular intervals, and named with the same criteria as described in section “Loading an Image in NEMO.” After selecting a slice to be analyzed, the metrical features relevant for the characterization of slice topology are directly extracted through the GUI (Figure 3).

The variables chosen in this analysis are useful for quantifying cell migration over time. After a pre-processing step in which images of slices are converted to binary images, some quantitative topological features are computed automatically:

- *Mean Fluorescence Intensity*: which is useful to quantify bleaching or expression of fluorescent proteins;
- *Distanc*e: external distance between the two opposite layers of cells within a segment;
- *Length*: two measurements related to the lengths of the two layers of the segment;
- *Average width*: two measurements related to the mean width of the two layers of the segment;
- *Min Width*: two measurements related to the minimum width of the two layers of the segment;
- *Max Width*: two measurements related to the maximum width of the two layers of the segment;
- *Linear Fit Error*: this gives a measure of the linearity of each layer.

All these parameters are collected in a datamatrix, with the same structure as described in section “Morphological Analysis,” which is automatically created when the user runs the topological analysis.

### Global datamatrix construction

In order to compare all the samples and to find analogies between the neurons analyzed, the morphological or topological parameters are assembled in a unique three-dimensional matrix structure, nominated “global datamatrix.” In this structure, data are organized into cells, variables and time; where *n* is the number of cells or slices, *m* the variables and *t* the number of days over which the images were obtained. Thus the final structure of the datamatrix is *n* × *m* × *t*.

The user can select the datamatrices of different neurons stored in the “Data Matrix” folder, and the global datamatrix is automatically created.

### Morphological and topological feature plots

After creating the three dimensional datamatrix, the user can plot the data. There is a specific command in the pop-up menu for plotting morphological or topological parameters stored in the datamatrix. The user can select the type of plot (day by day plots or time-dependent graphs) as well as the variables of interest. All the plots are automatically saved in a user specified directory.

### Three-way PCA

A novel feature of NEMO is the statistical analysis of the data extracted using the 3-way PCA, a multivariate technique. The 3-way PCA is a generalization of PCA, a popular technique that is often used for the exploratory analysis of a set of variables. While PCA analyzes data varying in two dimensions, 3-way PCA allows the analysis of sets of variables, associated with 3-way data sets, also called modes: variables, objects and conditions (in case of neurons photographed in different days of culture the conditions are the time intervals). This technique is aimed at transforming data to summarize the associated information in a small number of novel variables or principal components, able to express as much information as possible. Several models implement the 3-way PCA methodology (Kiers and van Mechelen, 2001). NEMO uses the Tucker3 model, an iterative technique first implemented by Leardi et al. (Leardi et al., 2000). In order to perform the 3-way PCA, the data need to be to re-organized. Considering the morphological features, from the global data matrix, with dimension *n* × *m* × *t*, *t* matrices with dimensions *n* × *m* are extracted. Then, the matrices are vertically concatenated. The result is a datamatrix with structure (*n* × *t*) × *m*. Formally, it is a two-dimensional matrix, but contains the entire dataset of information. This datamatrix is automatically saved in a user-specified folder.

In order to have a more efficient interpretation of the 3-way PCA, only the most significant morphological variables indicated in Table 1 are selected (Billeci et al., 2009).

After selecting the datamatrix to be analyzed, an input box is displayed, where the user can indicate the option to pre-process the data through a scaling to remove standardization offsets. The number of conditions (i.e., days of cell observation) and number of iterations also need to be indicated.

## Materials and methods

### Culture preparation and microscopy

Here we briefly describe a study to illustrate an application of NEMO. The software was used for the morphological studies of Purkinje neurons in culture and for comparison of two different genotypes. Purkinje cells expressing GFP were extracted from L7GFP WT mice and L7GFP/EN2+/−. The homeobox transcription factor, ENGRAILED 2 (EN2) is significantly associated with autism (Benayed et al., 2005) and has a role in both the embryonic and postnatal development of the mouse cerebellum (Millen et al., 1994). EN2+/− transgenic mice, heterozygote for EN2, have been developed and they display a phenotype reminiscent of the cerebellar anatomical abnormalities reported in autism. In L7GFP mice the expression of GFP in Purkinje is specifically driven by the Pcp-2 promoter (Zhang et al., 2001).

The culture preparation is described in Billeci et al. (2009). Two cultures for each of the two samples were used and fixed at different time points (8, 10, 14, and 15 days for WT; 10 and 15 days for EN2+/−). A total of 7 neurons from WT mice cultures and 5 neurons from EN2+/− autism model were analyzed. The cells were observed and photographed using a fluorescent microscope (Olympus, Italy) with a ×20 objective interfaced with a digital camera (Alkeria, Pisa).

### Neuron analysis with NEMO

All the images of Purkinje cells were first pre-processed in NEMO in order to obtain a skeleton of the neurons. Then the morphological analysis tool was applied to extract the morphological features of interest. The variables were extracted for all the cells at the different time points and visualized and compared using the graphical tool. Finally the mean values of all the cells analyzed for the two different types of mice at each time point were considered. All the lateral dimensions are represented in terms of pixels, and so all the values in the figures are expressed in this unit. At the magnification used (×20), one pixel corresponds to 0.182 μm.

### Statistical analysis

Before applying the 3-way PCA on the datamatrix, a matrix suitable for the multivariate technique was constructed. The structure was (12 × *t*) × 8, where the total number of cells analyzed is 12 (we used samples from both the cultures), the morphological variables selected for the 3-way PCA (Table 1) were eight and the conditions were the different days of culture, *t*. In order to compare the two cultures only the days of culture in common were selected: day 10 and 15. After realizing the datamatrix, the 3-way PCA tool was used to perform the statistical analysis of variables.

## Results

### Morphological analysis

The analysis of the morphological features extracted in NEMO enabled the identification of similarities and differences between the two types of cultures. Table 2 summarizes the results of the analyses at single time points.

**Table 2:** **Comparison of features extracted from image, Sholl and fractal analysis between Purkinje neurons from L7GFP WT and L7GFP/EN2+/− mice**.

**Variable**	**Neurons from L7GFP WT**	**Neurons from L7GFP EN2+/−**	**Comments**
Plot of the number of intersections vs. r	Gaussian	Maximum at r small and smaller at r large	Dendrites localized in the center in WT and near the soma in EN2+/−
Plot of log-log semi-log method	Log-log is a better approximation	Log-log is a better approximation	
Value of Δ	<1	<1	
Plot of minimum length vector	A lot of high peaks and higher value	Very small peaks	The principal axis is less tortuous and smaller in EN2+/−
Plot of angles between minimum length vector	A lot of high peaks	Very small peaks	Confirms that the principal axis is less tortuous in EN2+/−
Fractal dimension	1.55 ± 0.07	1.45 ± 0.12^*^	The fractality is lower in cells of EN2+/−

* p < 0.05 with respect to WT neurons.

**Figure A1:**
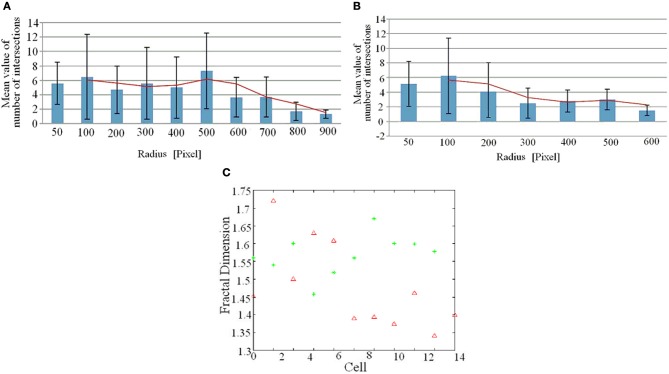
**Histogram of the mean values of number of intersections of all the Purkinje neurons from (A) WT mice vs. radii at 10 days, (B) EN2+/− mice vs. radii at 10 days. (C)** Value of fractal dimension for WT Purkinje neurons (green, asterisks) and EN2+/− neurons at 10 days (red, triangles).

The analysis of intersections showed that the trend of intersections vs. radius is Gaussian only in WT mice, with a peak corresponding to intermediate values of the radius. In EN2+/− the peak of intersections is shifted toward lower values of the radius (see Figures A1A, A1B in “Appendix”). The plot of log-log and semi-log methods revealed that both in WT and EN2+/− the log-log relationship better approximates the values of intersections. This result was confirmed by the value of Δ which is less than 1 for most of the cells, although the difference between the number of cells for which Δ is less than 1 and greater than 1 in the case of EN2+/− is less significant.

The analysis of the mean vector lengths on the principal axis and of angles between these vectors revealed that plots of EN2+/− cells have very smaller peaks with respect to WT cells.

The values of fractal dimension for all the cells of the two cultures were between 1.4 and 1.7, (see Figure A1C in “Appendix”) however, the mean fractal dimension of cells from EN2+/− is lower than the mean value of cells from WT mice (1.45 vs. 1.55, Table 2).

When the time dependent behavior of the two cultures was analyzed, we observed different trends, the most significant of which are summarized in Table 3. Interestingly the EN2+/− cells are consistently smaller, less arborized and die earlier than WT cells.

**Table 3:** **Comparison of the evolution in time of mean features extracted from image, Sholl and fractal analysis between Purkinje neurons from L7GFP WT and L7GFP EN2+/− mice**.

**Variable**	**PCs from L7GFP WT**	**PCs from L7GFP EN2+/−**	**Comments**
Maximum number of intersections	Higher value, peak at day 15	Lower value, peak at day 10	Less arborized dendrites in EN2+/−
Maximum radial extension	Higher value, peak at day 15 (104.6 ± 22.6 μm)	Lower value, peak at day 10 (58.4 ± 17.9 μm)^*^	Cells of EN2+/− grow less and die earlier
Maximum principal axis length	Higher value, peak at day 15 (102.4 ± 24.3 μm)	Lower value, peak at day 10 (49.3 ± 15.7 μm)^*^	Cells of EN2+/− grow less and die earlier
Cone angle	Same	Same	
Maximum soma size	Decreases in time (525.98 ± 127.1 μm^2^)	Constant, smaller (476.29 ± 201.5 μm^2^)	Cells of EN2+/− are smaller and less directional
Fractal dimension	Constant (1.55 ± 0.01)	Constant (1.43 ± 0.05)^*^	Cells of EN2+/− are less fractal

* p < 0.05 with respect to WT neurons.

### Comparative analysis

**Figure 4:**
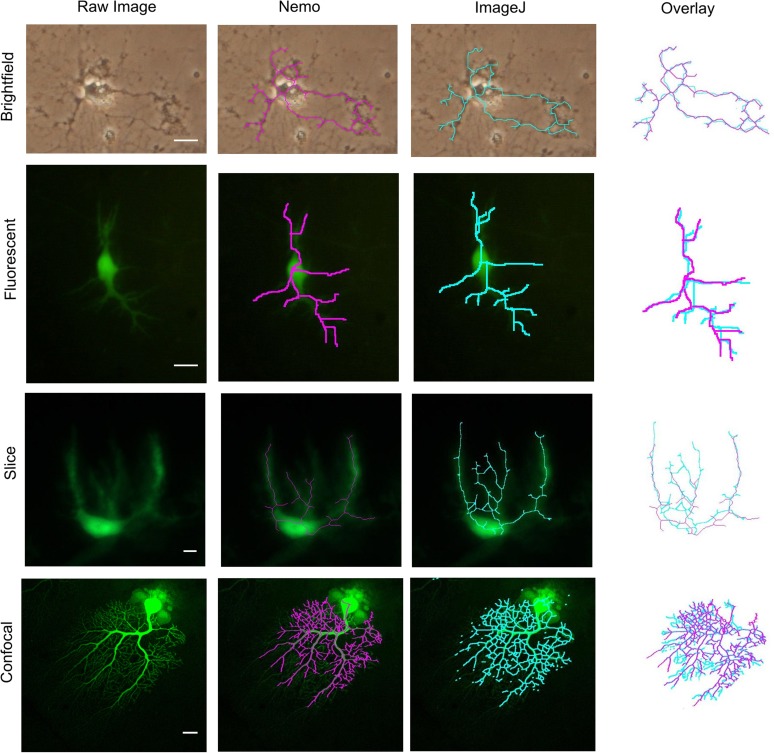
**Reconstruction of an isolated mice Purkinje neuron skeletons using NEMO and ImageJ.** Brightfield: CD1 mouse neonate 1 day after birth (P1); Fluorescent: L7GFP neonate P1; Organotypic slice: L7GFP P7-10 neonate; Confocal: Purkinje neuron from mouse cerebellum injected with Lucifer Yellow (Martone et al., 2002). Scale bar 10 μm. Comparative morphometric and Sholl parameters are provided in the “Appendix.” Image sources are cited in Acknowledgments.

We also compared the fidelity of the skeletonization process using both NEMO and ImageJ and the morphological and Sholl parameters output by NEMO and NeuronMorpho with ImageJ on four representative images of Purkinje neurons from different sources [respectively a bright field image, a fluorescence micrograph, an organotypic slice and a confocal image (Martone et al., 2002)]. The process was faster with NEMO due to the semi-automated tracing of neurite pathways (Figure 4). Although there were evident differences in the skeletons, particularly in the slice and confocal images, the morphological parameters measured in NEMO are similar to those extracted by NeuronMorpho, with no significant or systematic differences between the two (typically 3%, see “Appendix”). The Sholl parameters measured using NEMO and ImageJ's Advanced Sholl Analysis parameters plug-in were also similar and are provided in the “Appendix.” The results underline the well-known difficulty in neuron reconstruction, particularly with low resolution images, but suggest that quantification of discrete parameters may not be entirely reconstruction dependent.

### 3-way PCA analysis

**Figure 5:**
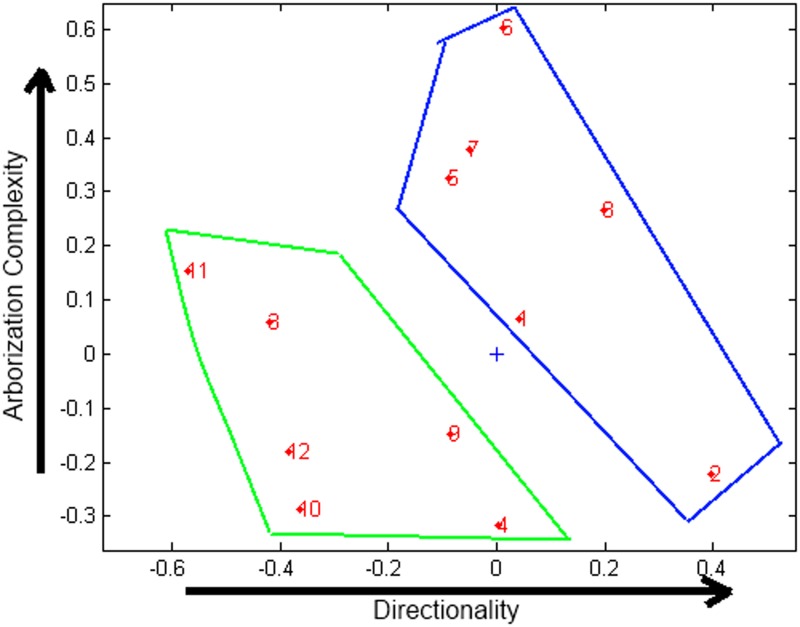
**Plot of objects obtained with 3-way PCA.** Green cluster: neurons extracted from EN2+/− mice, blue cluster: neurons extracted from WT mice. “+” indicates the axis origin.

The 3-way PCA tool delivers three plots: one for objects (cells), one for variables (morphological features) and one for conditions (days). In Figure 5 the most relevant graph for our analysis, that is the plot of objects, is shown. Two clusters, corresponding to the neurons from the two different cultures analyzed can be identified.

In order to properly interpret 3-way plots, it is essential to attribute appropriate identities to the axes. An analysis of the plots suggests that the *x*-axis represents the directionality of the cell, and *y*-axis the complexity of arborization, as represented in Figure 5. On the basis of the interpretation given to the axes, it can be concluded that neurons extracted from EN2+/− mice (green cluster) show less complexity in dendritic arborization and less cell directionality than neurons from WT mice (blue cluster). This result is in agreement with the conclusion obtained from the morphological analysis, as well as on studies of autistic subjects (Fatemi et al., 2002).

## Discussion

Morphometric analysis is relevant to the study of lifespan alterations in the neuritic field of neurons or neuronal morphological correlates of diseases, as well as the morphological implications of neurons under experimental conditions or the structure–function relationships in dendritic trees (White, 2007; Zhao et al., 2007; Brown et al., 2008). In order to analyse microscopic alterations over time in cell cultures or in brain tissue slices, it is important to have accurate, reliable, and reproducible measurements, which are not prone to human bias. Given that current imaging methods are able to provide high-resolution data in both space and time, in the last 20 years several commercial and open-source tools for morphological analysis and extraction of quantitative information on cell structure from microscopy images have been developed. Table 4 lists the features of the principal software available for microstructural and morphological analysis. From the table it is clear that not all the software are capable of a complete and exhaustive analysis: in fact, some tools are only dedicated to image pre-processing, or able to extract only few morphological parameters. Furthermore, many routine operations require significant manual intervention and interpretation, such as the tracing of each branch of the neurons with a pencil tool or mouse. In fact, most software do not implement automated or semi-automated image processing for parameter extraction. Most of the available software tools focus on the analysis of single parameters and many routine operations require significant manual intervention and interpretation. For example, in Neurolucida (Micro-BrightField, Williston, VT) (Glaser and Glaser, 1990), a commercial software for morphometric analysis of neurons used successfully by several investigators (Gianola and Rossi, 2001), the user must trace each neuron branch with a pencil tool or mouse. Moreover some of the tools are not open source, so that it is impossible to customize them for one's own purpose. Finally, some software require a commercial license and may be out of reach to scientists in developing and emerging countries.

**Table 4:** **Characteristics of the principal software for neuron morphological analysis**.

**Software**		**Sholl analysis**	**Fractal dimension**	**Soma area**	**Neurite length**	**Neuron count**	**Multivariate analysis**	**Principal advantages**	**Principal defects**
Neurolucida (Glaser and Glaser, 1990)		Yes	No	No	Yes	Yes	No	Good resolution	Not free; Only Windows
Syn-D (Schmitz et al., 2011)		Yes	No	Yes	Yes	Yes	No	Free; Automated; Time-efficient; Precise	Only Windows and MacOS
ImageJ *(Rasband, 1997–2012)*	NeuronJ (Meijering et al., 2004)	No	No	No	Yes	No	No	Free	Only tracing, no full analysis
	NeuriteTracer (Pool et al., 2007)	No	No	No	Yes	No	No	Free; Totallyautomated	Only tracing, no full analysis
	NeurphologyJ (Ho et al., 2011)	No	No	Yes	Yes	No	No	Free; Automated; Time-efficient	Incomplete analysis
	NeuronMetrics (Narro et al., 2007)	No	No	Yes	Yes	No	No	Free; Semi-automated	Incomplete analysis
	Sholl Analysis (Sholl, 1953)	Yes	No	No	Yes	No	No	Free; Automated	Only Sholl Analysis
	FracLac (Karperien, 2007)	No	Yes	No	No	No	No	Free; Automated	Only Fractal Dimension
	NeuronMorpho (Brown et al., 2005)	No	No	No	Yes	No	No	Free	Not Automated
NeuronStudio (Wearne et al., 2005)		Yes	No	No	Yes	No	No	Free; 3D reconstruction	Only high contrast images with fluorescent labels
Macro in MetaMorph (Gensel et al., 2010)		Yes	No	Yes	Yes	No	No	Accurate	Not free
TREES toolbox (Cuntz et al., 2010)		No	No	No	Yes	No	No	Good neurite reconstruction	Only tracing
IMARIS-Software *(Bitplane)*		No	No	No	Yes	Yes	No	4D real-time data visualization and analysis	Not specific for neurons
NEMO		Yes	Yes	Yes	Yes	Yes	Yes	Free; Accurate; Multivariate analysis	Preliminary folder organization

NEMO is an open source software provided with all the tools that can be useful for the analysis of neuron morphology. It consists of a set of computation algorithms written in Matlab and implemented in a GUI framework, in which it is easy to access the data and have a global view of the results. With respect to the other software listed in Table 4, its unique features are the automation of the method of data extraction from time lapse sequences of images and the use of the 3-way PCA for data analysis and classification. With the other tools, images need to be opened one at a time, traced, and then data are collected and saved separately. When dealing with multiple images of the same cell tracked over time, the procedure takes a considerable amount of effort. On the other hand, NEMO allows all the morphological features of interest and their variation in time to be obtained in a single operation. The 3-way PCA can be used to unravel correlations between significant morphometric variables in neurons and for the classification of cells according to their morphology.

NEMO has been implemented focusing on the application for dynamic analysis of neurons. The batch processing feature allows all the metrical variables which characterize cell morphology and their variation in time to be obtained in a single operation. Once the software is run, a global flow of operations begins so as to obtain all the parameters for a given cell at different times. In the same modality topological features as well as the number of neurons present in images of brain slices photographed at different time points can be extracted. All the information is then saved in a single matrix (the datamatrix) from which selected parameters can be plotted. The concatenation of matrices from different cells then allows graphical analyses and comparisons between different groups and parameters.

In this work NEMO was applied to a comparative study of neurons in culture obtained from labeled EN2+/− mice, an animal model of autism, and labeled WT mice. The results show that the EN2+/− cells are consistently smaller, less arborized and die earlier than WT cells. This finding correlates well with autoptic studies (Fatemi et al., 2002; Courchesne et al., 2005), which have shown that autistic brains have fewer Purkinje cells than normal brains and merits further investigations.

NEMO is proposed here as an alternative and novel open-source software for the morphometric characterization and classification of neurons and brain slices. The software was developed out of necessity as there are currently no tools available for handling and processing large quantities of data on single cells as they evolve over time. NEMO could be of interest and use to researchers involved in morphometric quantification and advanced statistical analysis; the more unique features have been implemented as ImageJ plug-ins.

### Conflict of interest statement

The authors declare that the research was conducted in the absence of any commercial or financial relationships that could be construed as a potential conflict of interest.

## Appendix

**Table A1:** **Comparative morphometric analysis using NEMO and ImageJ's NeuronMorpho and Advanced Sholl analysis plug-ins**.

**Brightfield image (275 pixels —50 μm)**				**Fluorescent Image (275 pixels —50 μm)**				**Organotipic slice (154 pixels —50 μm)**				**Confocal image (417 pixels —50 μm)**			
**NEMO**		**IMAGEJ**		**NEMO**		**IMAGEJ**		**NEMO**		**IMAGEJ**		**NEMO**		**IMAGEJ**	
**Soma Area (μm^2^)**				**Soma Area (μm^2^)**				**Soma Area (μm^2^)**				**Soma Area (μm^2^)**			
63.14		66.44		129.58		126.28		279.34		260.37		175.68		177.98	
**Extension (μm)**				**Extension (μm)**				**Extension (μm)**				**Extension (μm)**			
54.86		55.04		66.09		65.80		78.79		81.58		73.42		73.14	
**Sholl analysis**				**Sholl analysis**				**Sholl analysis**				**Sholl analysis**			
**Radii (μm)**	**Inter-section**	**Radii (μm)**	**Inter-section**	**Radii (μm)**	**Inter-section**	**Radii (μm)**	**Inter-section**	**Radii (μm)**	**Inter-section**	**Radii (μm)**	**Inter-section**	**Radii (μm)**	**Inter-section**	**Radii (μm)**	**Inter-section**
3.83	2	3.83	2	3.93	2	3.93	2	7.35	4	7.35	3	0.79	2	0.79	2
9.58	5	9.58	7	8.49	5	8.49	5	14.29	5	14.29	4	4.76	2	4.76	4
15.32	12	15.33	11	13.04	4	12.98	4	21.23	5	21.23	4	8.72	14	8.72	15
21.07	20	21.08	20	1.60	6	17.59	7	28.17	5	28.17	5	12.69	22	12.69	20
26.82	16	26.83	13	22.15	4	22.15	4	35.12	6	35.12	6	16.65	19	16.65	19
32.57	13	32.58	14	26.70	3	26.70	3	4206	4	42.06	4	20.61	20	20.61	15
38.32	12	38.33	12	31.26	3	31.25	3	49.01	3	49.00	3	24.60	18	24.58	11
44.07	6	44.08	6	35.81	2	35.81	2	55.94	3	55.94	3	28.54	6	28.54	6
49.82	6	49.90	5	40.37	1	40.36	1	62.88	2	62.88	2	32.51	5	32.51	5
55.57	1	55.58	1					69.82	1	69.82	1				
Max number of intersections				Max number of intersections				Max number of intersections				Max number of intersections			
20		20		6		7		6		6		22		20	

The data refer to the neurons in the manuscript's Figure 4.

**Table A2:** **Results of Sholl analysis between Purkinje neurons from L7GFP WT and L7GFP EN2+/− mice at day 10 of culture**.

**Radius (Pixel)**	**Number of intersections (mean +/− s.d)**	
	**L7GFP WT**	**L7GFP EN2+/−**
50	5.50 ± 3.00	5.00 ± 3.00
100	6.25 ± 5.9	6.00 ± 5.3
200	4.75 ± 3.37	8.25 ± 4.12
300	5.75 ± 5.00	2.25 ± 1.87
400	5.00 ± 3.25	3.5 ± 1.6
500	7.25 ± 5.12	2.75 ± 1.25
600	3.75 ± 2.6	1.75 ± 0.8
700	3.75 ± 2.6	−
800	1.75 ± 1.25	−
900	0.5 ± 0.5	−
